# Metabolomic profiling of tumor-infiltrating macrophages during tumor growth

**DOI:** 10.1007/s00262-020-02622-8

**Published:** 2020-06-09

**Authors:** Naoki Umemura, Masahiro Sugimoto, Yusuke Kitoh, Masanao Saio, Hiroshi Sakagami

**Affiliations:** 1 Department of Oral Biochemistry, Asahi University School of Dentistry, Gifu, 501-0296 Japan; 2 Health Promotion and Preemptive Medicine, Research and Development Center for Minimally Invasive Therapies, Institute of Medical Sciences, Tokyo Medical University, Tokyo, Japan; 3 Institute for Advanced Biosciences, Keio University, Yamagata, Japan; 4 Department of Pathology and Translational Research, Gifu University Graduate School of Medicine, Gifu, Japan; 5 Graduate School of Health Sciences, Gunma University, Maebashi, Gunma Japan; 6 Meikai University Research Institute of Odontology (M-RIO), Saitama, Japan

**Keywords:** Myeloid derived suppressor cells, Tumor infiltrating macrophages, Metabolomics, Tumor necrosis factor-alpha

## Abstract

Myeloid-derived suppressor cells (MDSCs) and tumor-associated macrophages (TAMs) are both key immunosuppressive cells that contribute to tumor growth. Metabolism and immunity of tumors depend on the tumor microenvironment (TME). However, the intracellular metabolism of MDSCs and TAMs during tumor growth remains unclear. Here, we characterized CD11b+ cells isolated from a tumor-bearing mouse model to compare intratumoral TAMs and intrasplenic MDSCs. Intratumoral CD11b+ cells and intrasplenic CD11b+ cells were isolated from tumor-bearing mice at early and late stages (14 and 28 days post-cell transplantation, respectively). The cell number of intrasplenic CD11b+ significantly increased with tumor growth. These cells included neutrophils holding segmented leukocytes or monocytes with an oval nucleus and Gr-1^hi^ IL-4Rα^hi^ cells without immunosuppressive function against CD8 T cells. Thus, these cells were classified as MDSC-like cells (MDSC-LCs). Intratumoral CD11b+ cells included macrophages with a round nucleus and were F4/80^hi^ Gr-1^lo^ IL-4Rα^hi^ cells. Early stage intratumoral CD11b+ cells inhibited CD8 T cells via TNFα. Thus, this cell population was classified as TAMs. Metabolomic analyses of intratumoral TAMs and MDSC-LCs during tumor growth were conducted. Metabolic profiles of intratumoral TAMs showed larger changes in various metabolic pathways, e.g., glycolysis, TCA cycle, and glutamic acid pathways, during tumor growth compared with MDSL-LCs. Our findings demonstrated that intratumoral TAMs showed an immunosuppressive capacity from the early tumor stage and underwent intracellular metabolism changes during tumor growth. These results clarify the intracellular metabolism of TAMs during tumor growth and contribute to our understanding of tumor immunity.

## Introduction

Previous studies have shown that host immune mechanisms are not entirely effective against tumors, probably due to the acquisition of immune escape mechanisms by tumor cells or T cell intolerance in tumor tissue [1–3]. Myeloid-derived suppressor cells (MDSCs) and/or tumor-associated macrophages (TAMs) are strongly associated with tumor immunosuppression and affect systemic immunity and induce cancer cachexia [4, 5]. The numbers of both MDSCs and TAMs in tumor tissues correlated with poor prognosis of cancer patients [6–9]. MDSCs and TAMs constitute the majority of immunocompetent cells that exhibit immunosuppressive functions in tumors [10].

MDSCs are derived from immature bone marrow cells that are induced by various cytokines and soluble factors under pathological conditions such as infection, cancer, sepsis, trauma, bone marrow transplantation, and several autoimmune diseases. These cells accumulate in lymphatic tissues and blood and show pleiotropic immunosuppressive activities [11–13]. In the tumor microenvironment, cancer cells secrete various molecules involved in the accumulation and recruitment of MDSCs from immature bone marrow cells. These molecules include granulocyte-macrophage colony-stimulating factor (GM-CSF), macrophage colony-stimulating factor (M-CSF), transforming growth factor (TGF-β), tumor necrosis factor (TNF)-α, vascular endothelial growth factor (VEGF), prostaglandin E2, cyclooxygenase 2 (COX2), S100A9, S100A8, interleukin (IL)-1β, IL-6, and IL-10 [14–16]. MDSCs are also indirectly involved in the immunosuppression network in the TME during tumor growth. MDSCs strongly potentiate neoangiogenesis by the production of matrix metalloproteinases (MMPs) and VEGF mediated by hypoxia-inducible factor α (HIF-1α) under hypoxic conditions [17]. MDSCs can also eliminate nutrients necessary for T cell function, such as L-arginine, via activation of arginase-1 (Arg-1) activity [18]. MDSCs also deplete L-cysteine by limiting the supply of amino acids necessary for lymphocyte activity [19]. Moreover, MDSCs are thought to exert immunosuppression via reactive oxygen species (ROS) and generate nitric oxide (NO) via inducible nitric oxide synthase (iNOS) by the generation of NADPH oxidase, resulting in inducing T cell suppression and apoptosis [18]. Together these findings demonstrate that MDSCs induce T cell suppression and apoptosis.

Macrophages with various phenotypes are observed in tumor tissues [20]. TAMs show two important characteristics. First, TAMs highly express Arg1, VEGF, osteopontin, MMPs, IL-10, TGFβ, and TNFα and thus inhibit the anti-tumor immune response [21, 22]. Second, TAMs are regulated by DNA damage response 1 (REDD1), and as a result TAMs regulate angiogenesis and neoangiogenesis [23]. Previous studies suggested that MDSCs exposed to tumor cells or tissues differentiate into TAMs with tumor immunosuppressive function [10, 24]. However, to the best of our knowledge, the differentiation and changes in the immunosuppressive capacity of TAMs and MDSCs in the TME during tumor growth are unknown.

We previously analyzed CD11b+ cells as an antigen of monocytes/macrophages and isolated these cells from tumor tissue in an isogenic graft mouse model. CD11b+ cells in tumor tissue represented a F4/80^hi^ cell population and expressed CXCL10 and CD206; this cell population inhibited CD8 T cell proliferation [25]. Although the characteristics of TAMs in tumor tissue and the tumor immunosuppressive functions have been clarified [26], few studies have examined the correlation between MDSCs and TAMs in tumor immunosuppression.

Various specific intercellular metabolic changes, such as upregulated glycolysis (Warburg effect), have been identified in cancer cells. Research in tumor immunology has demonstrated that intracellular metabolic abnormalities of immune cells in cancer conditions are associated with tumor growth and immune suppression, such as the immunosuppressive mechanism of T cells by the intracellular metabolic change [27, 28]. However, the precise relationship between immunosuppression during tumor growth and these metabolic changes remain unclear. Furthermore, the changes in intracellular metabolic pathways in MDSCs in the systemic circulation and TAMs in tumor tissue accompanying tumor growth have not been clarified. Better understanding of these mechanisms would contribute to establishing effective immunotherapies.

Here, we utilized an isogenic graft mouse model to obtain CD11b+ cells in the tumor or spleen and analyzed cell antigen markers, immunosuppressive functions, and intracellular metabolism with the monocyte lineage marker during tumor growth. We investigated the tumor immune mechanism of intrasplenic MDSCs and intratumoral TAMs by exploring changes in intracellular metabolism during tumor growth.

## Methods

### Mice

Male 6- to 8-week-old C57BL/6 mice were purchased from Sankyo Lab Service Corporation (Tokyo, Japan). TNFα knockout mice (TNF KO mice) in the B6 background were purchased from The Jackson Laboratory (Bar Harbor, MA, USA). The mice were maintained in accordance with the guideline of the committee on animals of Meikai University School of Dentistry. This study was approved by the animal ethics committee of Meikai University (No. A-1510).

### Cell culture

The murine colon adenocarcinoma (MCA) 38 cell line was provided by Dr. Yang Liu (Ohio State University, Columbus, OH, USA). The cells were cultured at 37 °C in a humidified 5% CO_2_ atmosphere in Roswell Park Memorial Institute (RPMI) 1640 media (Invitrogen Life Technologies, Carlsbad, CA, USA) supplemented with 10% fetal bovine serum (FBS) (Nichirei Bioscience, Tokyo, Japan) and antibiotics (100 U/mL penicillin and 100 g/mL streptomycin).

### Implantation of tumor cells

MCA38 cells (1 × 10^6^) were resuspended in 0.1 mL PBS and subcutaneously injected into the lateroabdominal area of C57BL/6 mice or TNF KO mice. Tumor growth was monitored using 20 C57BL/6 mice. The tumor volume was calculated using the formula a × b^2^/2, in which ‘a’ is the tumor length and ‘b’ is the tumor diameter. No significant difference in body weight of the mice was observed between control mice without cell inoculation and tumor cell-inoculated mice. Tumors were palpable after day 14, and thus, the first 14 days after tumor inoculation were designated as the early stage. By four weeks later (day 28), the tumor volume exceeded 1,000 mm^3^ and this time point was considered the late stage. In another experiment, 20 mice were divided into four groups and inoculated as described above. At various time points (day 0, day 14, day 21, and day 28; n = 5/group), mice were killed and the number of splenic cells and splenic CD11b+ cells were determined.

### Preparation of tumor infiltrating cells and spleen cells

Five C57BL/6 mice were killed 14 and 28 days after tumor cell implantation. Tumor tissue was finely chopped and incubated with the enzyme mix from the tumor dissociation kit (MACS Miltenyi Biotec, Berdish-Gladbach, Germany) according to the manufacturer’s instruction. The sample was then applied to a MACS SmartStrainer (40 µm). Red blood cells in the sample were removed using a red blood cell lysis solution (MACS Miltenyi Biotec). For total spleen cell isolation, red blood cells were removed using the same procedures, and the cells were applied to a MACS SmartStrainer (40 µm). CD11b+ cells were isolated using anti-CD11b magnetic immunobeads according to the manufacturer’s instructions (MACS Miltenyi Biotec).

### Flow cytometric analysis

Cells were pre-incubated with 10 g/ml anti-CD16/32 antibody (4.2G2, PharMingen, San Diego, CA, USA) at 4 °C for 30 min before staining with specific antibodies. Via-Probe (BD Bioscience) was used for dead cell exclusion in all staining experiments. To analyze CD11b+ cell surface antigen expression, allophycocyanin (APC)-conjugated anti-CD11b (M1/70, PharMingen) and FITC-, PE-, or cytochrome-conjugated anti-F4/80 were used. Additional antibodies included FITC-conjugated anti-Ly6C (ER-MP20, BMA Biomedicals, Augst, Switzerland), PE-conjugated anti-Ly6G (Gr-1; RB6-8C5, PharMingen), biotin-conjugated anti-IL-4Rα (polyclonal goat IgG, R&D Systems, Inc., Minneapolis, MN, USA), and PE-conjugated anti-CD11c (clone HL3, PharMingen). FITC- or PE-conjugated hamster IgG (PharMingen), rat IgG1 (PharMingen), rat IgG2a (PharMingen and Serotec Ltd., Oxford, UK), rat IgG2b (PharMingen), and biotin-conjugated control goat IgG (R&D Systems, Inc.) served as control antibodies. All antibodies were used at 10 µg/ml. The cells were incubated with the antibodies for 30 min at 4 °C and then washed with PBS(-). If biotin-conjugated antibody was used, the samples were subsequently stained with avidin-conjugated PE (PharMingen) for 30 min at 4 °C and then washed with PBS. The samples were fixed with 1% paraformaldehyde/PBS(-) and analyzed using a FACSCalibur flow cytometer and CellQuest software (Becton Dickinson Japan, Tokyo).

### May-Giemsa staining

CD11b+ cells that were separated by the MACS magnetic system from either tumor-bearing spleen or intratumoral tissue were adhered to a glass slide by a Cytocentrifuge (Thermo Fisher Scientific K.K., Yokohama, Japan). The cell smears were prepared and stained first with May-Grunwald’s solution (Merck KGaA, Darmstadt, Germany) for 3 min and then with a dilution of May-Grunwald’s solution (1:1 in water) for 1 min. After washing with water, the cell smears were stained for 30 min with × 0.025 diluted Giemsa’s solution (Merck KGaA) diluted with 6.7 mM phosphate buffer, pH 6.4. The smear was then examined under a microscope.

### Evaluation of inhibition of cytotoxic T cell activity

CD8+ cells were isolated from spleens from C57BL/6 mice using anti-CD8 magnetic immunobeads (MACS Miltenyi Biotec) according to the manufacturer’s instructions. CD8+ cells were labeled by carboxyfluorescein diacetate succinimidyl ester (CFSE) from the Cell Trace™ CFSC Cell Proliferation kit (Thermo Fisher Scientific K.K.) according to the manufacturer’s instruction [29]. CFSE-labeled CD8+ cells were treated with 5 µg/mL concanavalin A (ConA) as a non-specific antigen. Cells were then co-cultured with CD11b+ cells that were isolated from tumor-bearing spleen or intratumoral tissue. At 24 h later, the cells were stained with PE-anti-CD8 (PharMingen) to identify T cells and CFSE was detected by 488 nm excitation using a FACS Calibur flow cytometer and CellQuest software (Becton Dickinson Japan).

### Co-culture of activated splenic cells with CD11+ cells from tumor-bearing mice

Splenic cells were isolated from a control mouse (2 × 10^5^ cells) and plated on CD3 10 μg/mL bound 96-well flat plates. CD28 (2 μg/mL) was added, and after 24 h, cells were co-cultured with CD11b+ cells (2 × 10^5^ cells) of tumor or spleen from tumor-bearing mice. After 24 h, the cells were harvested by trypsinization and then stained with PE-anti-CD8 and APC-anti-CD11b after Fc blocking by anti-CD16/32 antibody. The CD8 T cell population was detected by gating CD8-positive and CD11b-negative cells using a flow cytometer. For each experiment, splenic cells were isolated from one wild-type mouse. CD11b+ cells of the tumor or spleen were obtained from tumor-bearing mice 14 and day 28 days after tumor implantation; one mouse per group was used, and the isolation procedure was attempted three times.

### ^51^Cr release assay

Tumor-infiltrating CD11b+ cells isolated on MACS separation columns were cultured in RPMI-1640 medium containing 10% FBS overnight at 37 °C with 5% CO_2_ and used as effector cells. CD8+ cells isolated from C57BL/6 mouse spleen using anti-CD8 magnetic immunobeads (1 × 10^6^ cells) were labeled with 3.7 MBq Na_2_CrO_4_ for 1 h at 37 °C with 5% CO_2._ The labeled CD8+ cells were treated with 5 µg/mL ConA as non-specific antigen and used as target cells. The effector and target cells were combined at various ratios in 96-well U-bottom plates and incubated for 5 h at 37 °C with 5% CO_2_. Supernatants were then collected, and radioactivity was quantified with a γ counter. Spontaneous release was determined by incubation of target cells in the absence of effector cells, and maximum release was determined by incubation of target cells in 0.1% Triton X-100. Specific cytotoxicity was calculated as ^51^Cr release (%) = 100 × (cpm experimental − cpm spontaneous)/(cpm maximum − cpm spontaneous).

### Metabolomic analysis

Aliquots of CD11b+ cells from spleen or tumor site were stained with trypan blue and viable cell numbers were counted using a hemocytometer. Three mice per group were used on day 14 and 28 after tumor cell inoculation. The remaining cells were washed twice with 5 mL of ice-cold 5% D-mannitol and then immersed for 10 min in 1 mL of methanol containing internal standards (25 µM each of methionine sulfone, 2-[N-morpholinol]-ethanesulfonic acid and D-camphor-10-sulfonic acid). The methanol extract (supernatant) was collected. To 400 µL of the dissolved samples, 400 µL of chloroform and 200 µL of Milli-Q water were added and the mixture was centrifuged at 10,000 × g for 3 min at 4 °C. The aqueous layer was filtered to remove large molecules by centrifugation through a 5-kDa cut-off filter (Millipore, Billerica, MA, USA) at 9,100 × g for 2.5 h at 4 °C. Next, 320 mL of the filtrate was concentrated by centrifugation and dissolved in 50 mL of Milli-Q water containing reference compounds (200 mM each of 3-aminopyrrolidine and trimesate) immediately before capillary electrophoresis-time-of-flight-mass spectrometry (CE-TOF-MS) analysis.

The instrumentation and measurement conditions used for CE-TOF-MS are described elsewhere [30–32]. Briefly, positive and negative metabolites were independently measured in cation and anion modes.

### Data analysis and statistical analysis

For analyses of the number of splenic cells, data are presented as means ± standard deviations (SD) and were evaluated using one-way analysis of variance followed by Dunnett’s multiple comparisons test (day 0 vs. day 14, 21, or 28). For the detection of activated CD8 T cell population, one-way analysis of variance followed by the Tukey–Kramer test was performed to compare CD8 T cell populations in co-cultures of various conditions. For the ^51^Cr release analysis, Student’s *t*-tests (two-tailed) were performed to compare the data between TAMs and TNF-KO TAMs. Raw data from metabolomics analyses were analyzed using our proprietary software, MasterHands [33], including the detection of all possible peaks, elimination of noise and redundant features, and generation of an aligned data matrix with annotated metabolite identities [34]. Metabolites were identified with matched *m/z* and corrected migration times with our standards library. Concentrations were calculated using external standards based on relative area, i.e., the area divided by the area of the internal standards.

To compare the metabolic data between MDSCs and TAMs, Student’s *t*-tests (two-tailed) were used. To compare the data between 14 and 28 days post-cell transplantation, paired Student’s *t*-tests were used.

GraphPad Prism (Ver. 5.04, GraphPad Software Inc., San Diego, CA, USA) was used for statistical tests. Metabolites were visualized in our proprietary pathway visualization tool (Keio University, Yamagata, Japan) [35].

## Results

### Intrasplenic CD11b+ cell numbers increase with tumor growth

**Fig. 1:**
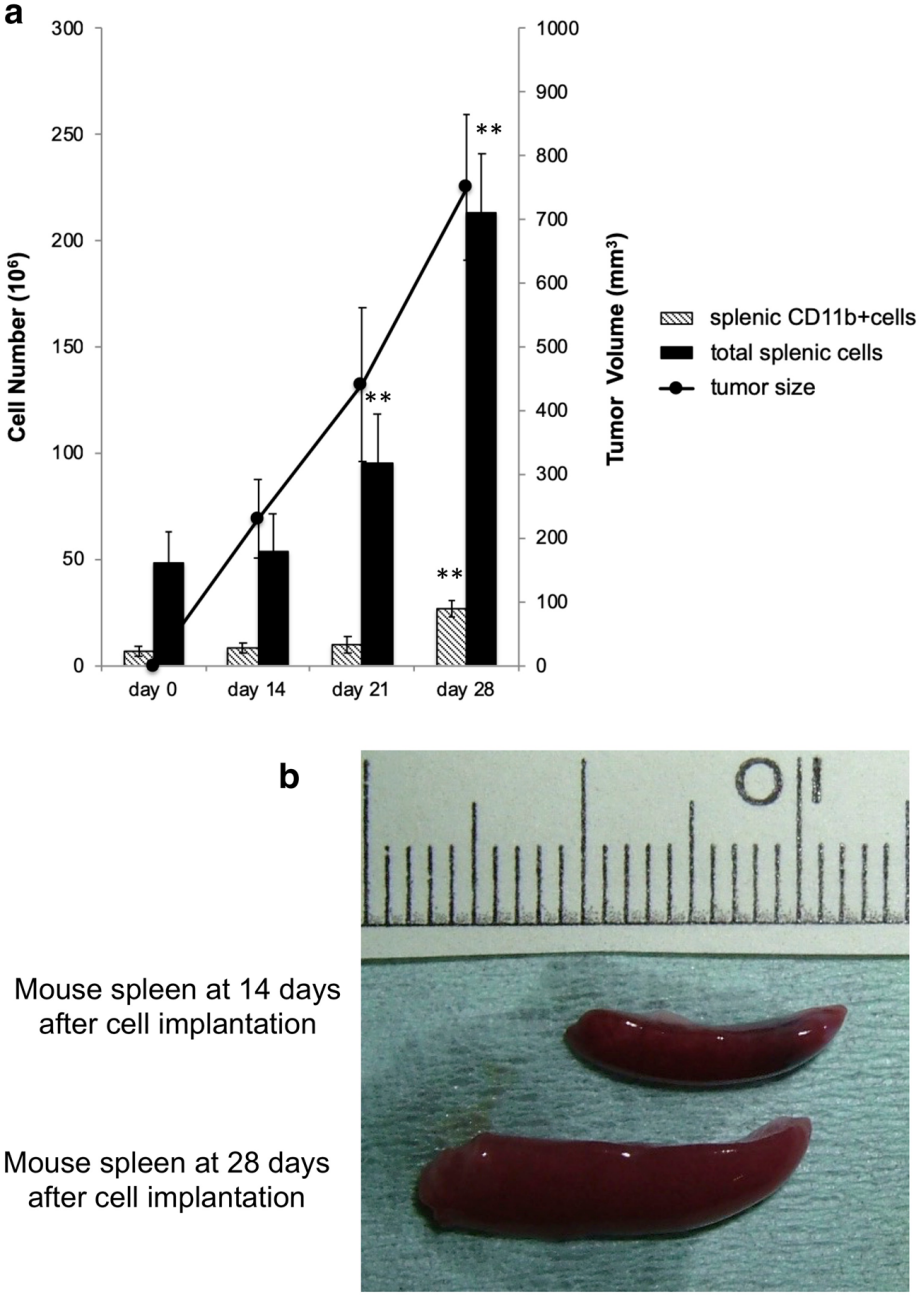
Characteristics of splenic CD11b-positive cells during tumor growth **a** Cell numbers of total splenic cells and splenic CD11b-positive cells (isolated by magnetic beads) from an isogenic graft mouse model during tumor growth. The line graph indicates the tumor volume. **b** Mouse spleen at 14 days and 28 days after cell implantation. *P < 0.05 and **P < 0.01; Dunnett’s test for day 0 vs. day 14, 21, or 28; days 0, 14, 21, and 28: n = 5/group

The relationship between the number of splenocytes and CD11b+ splenocyte cells with tumor growth in the isogenic graft mouse model was analyzed (Fig. 1a). In mice without tumors (day 0), the total number of splenocytes was approximately 48×10^6^ cells, among which 6.4×10^6^ were CD11b+ cells. In mice with tumors (day 21), the total number of splenocytes (9.5×10^7^ cells) was significantly increased compared with the total number of splenocytes in mice at day 0 (*P*<0.01). The tumor volume reached 750 mm^3^ on day 28, and the total number of splenocytes and CD11b+ cells was significantly increased to 2.13×10^8^ (*P*<0.01) and 2.66×10^7^ cells (*P*<0.01), respectively (Fig. 1a, b).

### Intratumoral CD11b+ cells inhibit CD8 T cell proliferation via TNFα

**Fig. 2:**
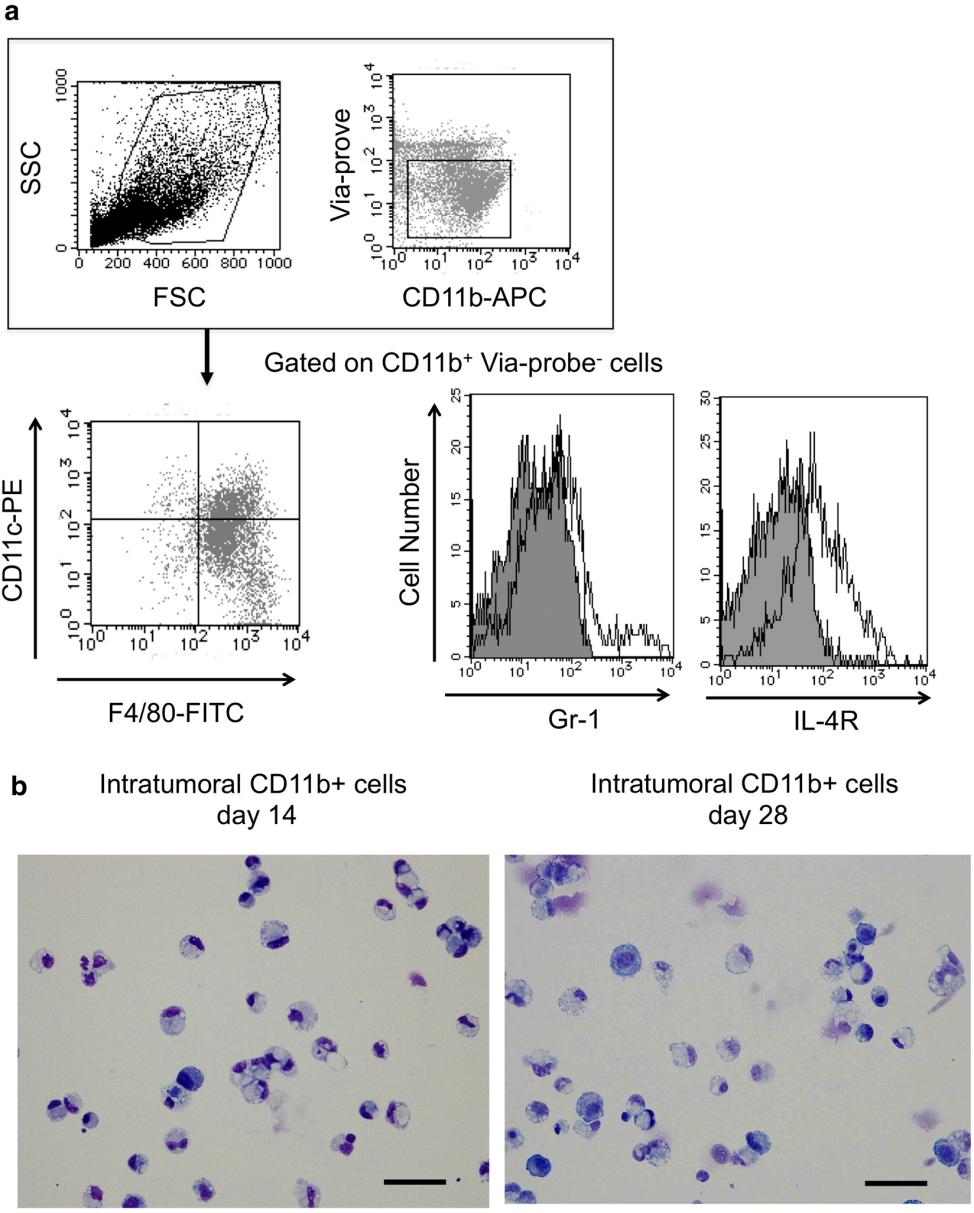

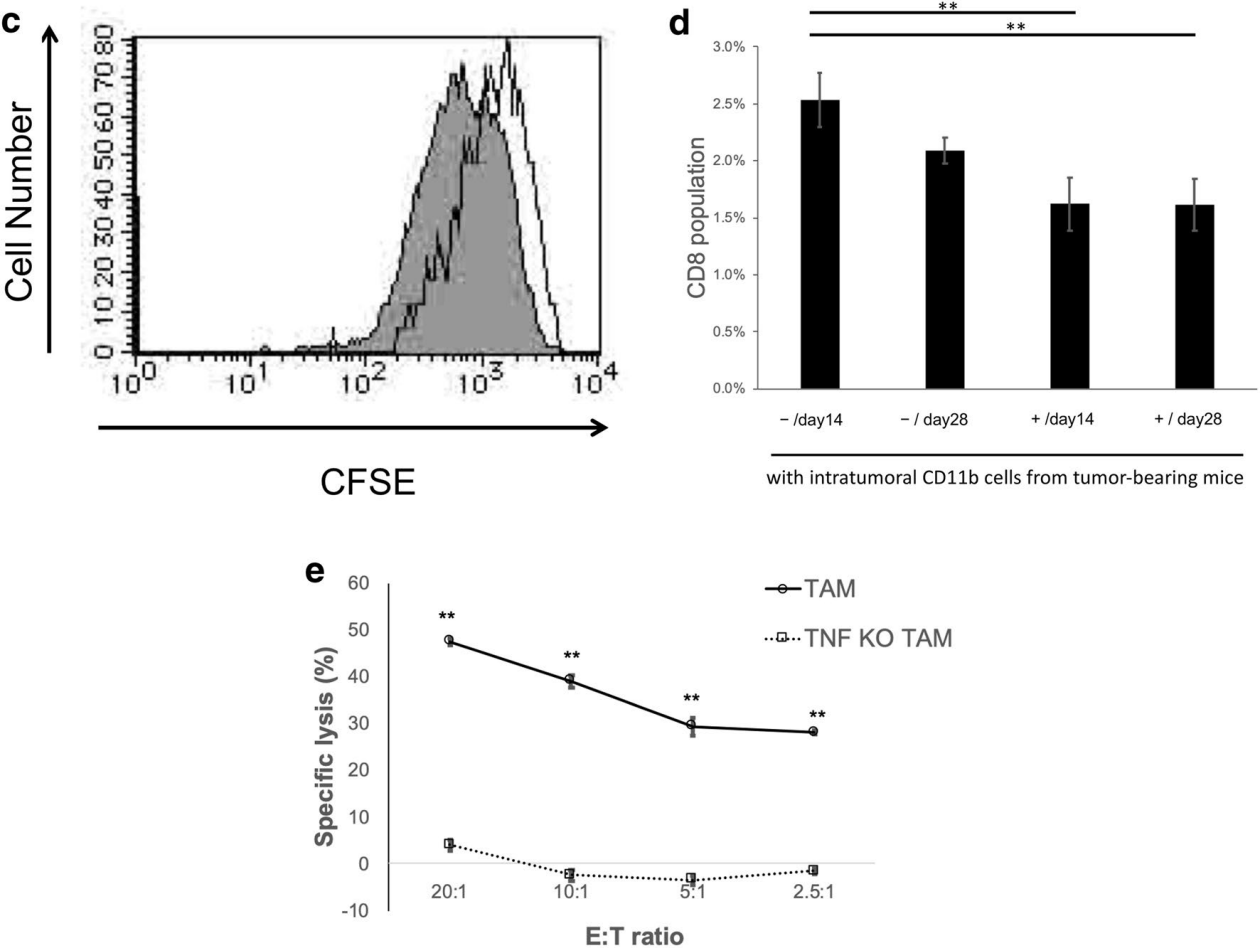
Intratumoral CD11b-positive cells inhibit CD8 cells **a** Flow cytometry analysis of intratumoral CD11b+ cells from 14 days after cell implantation. An example of gating used for the analysis is shown. First, the cell population was gated from side scatter (SSC)-forward scatter (FSC) (A, upper left). To eliminate dead cells, the cells were gated by Via-prove-negative and CD11b+ cells (A, upper right). The lower panels show results for F4/80, Gr-1, and IL-4Rα. **b** May-Giemsa staining of intratumoral CD11b+ cells at 14 days and 28 days after cell implantation. Magnification, × 400. The scale bar indicates 50 μm. **c** The CD8 inhibitory function assay of intratumoral CD11b+ cells from 28 days after cell implantation. CFSE-labeled CD8+ T cells stimulated with ConA were co-cultured with intratumoral CD11b+ cells from 14 days after cell implantation for 24 h. **d** The activated CD8 T cell population changes. Splenic cells unstimulated or stimulated by CD3 and CD28 were co-cultured with intratumoral CD11b+ cells from 14 or 28 days after cell implantation for 24 h.  − and + indicate unstimulated splenic cells and CD3/CD28-stimulated splenic cells from normal mice, respectively. *P < 0.05 and **P < 0.01 (Tukey–Kramer test). **e** ^51^Cr-release assay. Con-A-activated CD8 T cells labeled with Na_2_CrO_4_ and intratumoral CD11b+ cells were co-incubated for 5 h. Radioactivity was quantified with a γ counter, and specific cytotoxicity was calculated. *P < 0.05 and **P < 0.01 (Student’s *t*-test between TAMs and TNF KO TAMs). Representative data from three independent experiments that were repeated three times. One mouse per group was used 14 and 28 days after tumor implantation. One spleen of a normal mouse was used once, and the assay was repeated three times (**d**)

The phenotype of CD11b+ cells isolated from tumor tissue was analyzed. Intratumoral CD11b+ cells isolated in the early stage (14 days after tumor inoculation) were F4/80^hi^, Gr-1^lo^, and IL-4Rα^hi^. No difference in IL-4Rα expression in intratumoral CD11b+ cells was observed between early stage and late stage (28 days after cell inoculation) (Fig. 2a). Intratumoral CD11b+ cells in both early and late stage had a round nucleus and were predominantly endoplasmic reticulum-rich macrophages or monocytes with vacuoles with reduced endoplasmic reticulum compared with macrophages (Fig. 2b). Furthermore, intratumoral CD11b+ cells at early stage showed suppression of CD8 T cells (Fig. 2c–e). There was no significant difference in the suppression of CD8 T cells between early stage and late stage intratumoral CD11b+ cells (Fig. 2d). In contrast, intratumoral CD11b+ cells from TNFα KO mice did not show suppression of CD8 T cells (Fig. 2e). These data indicated that TNFα is important in intratumoral CD11b+ cells for the suppression of CD8 T cells from early stage. Based on these data, intratumoral CD11b+ cells were considered as TAMs.

### Intrasplenic CD11b+ cells show increased IL-4Rα but do not inhibit CD8 T cell proliferation

**Fig. 3:**
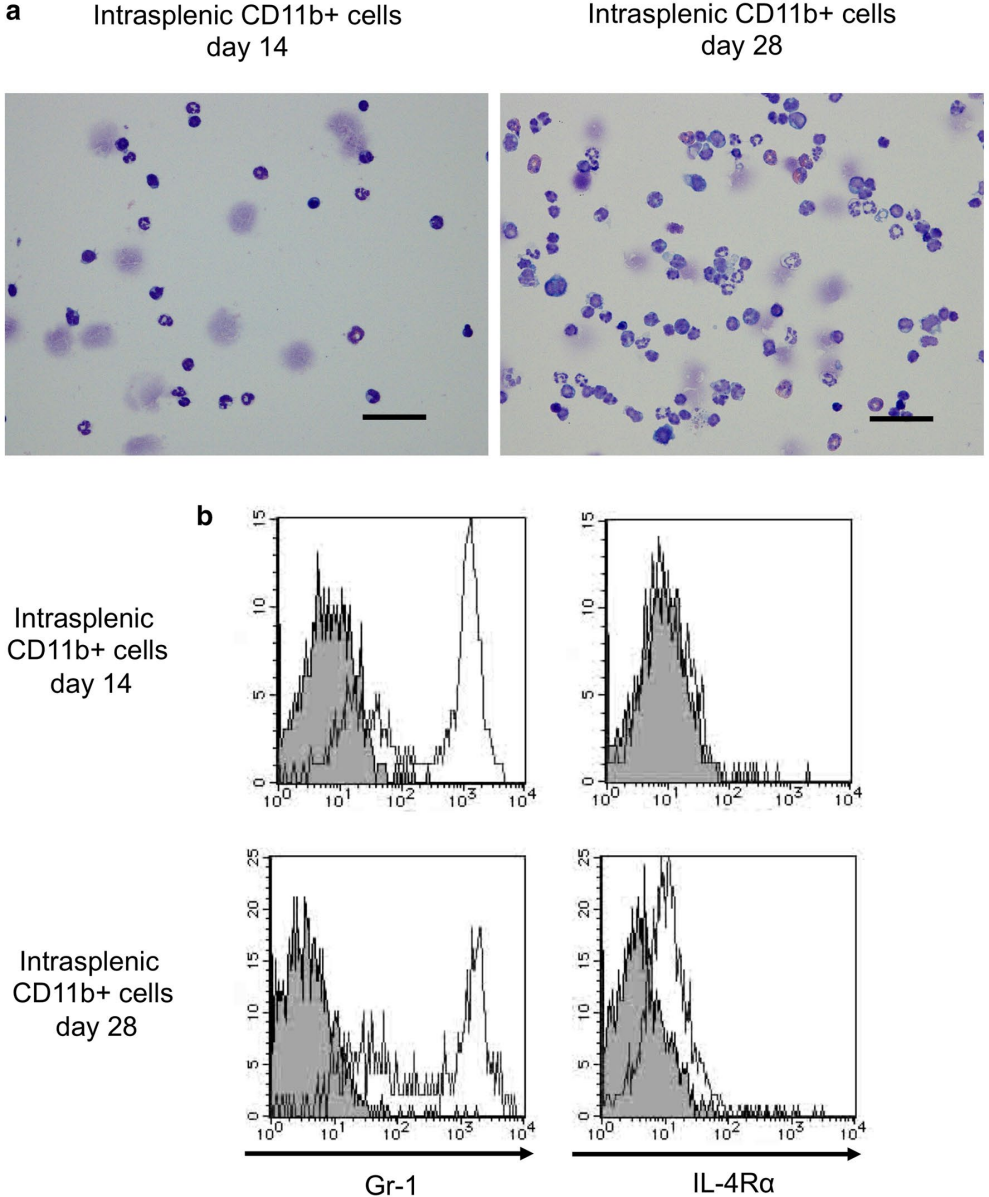

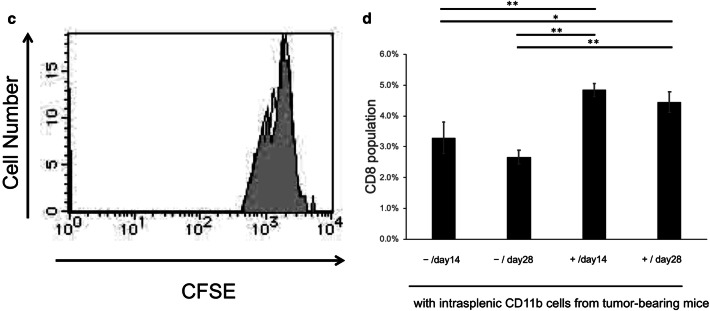
Intrasplenic CD11b-positive cells show increase IL-4Rα expression **a** May-Giemsa staining of CD11b-positive splenic cells that were isolated at 14 days and 28 days after cell implantation. Magnification, × 400. The scale bar indicates 50 μm. **b** Flow cytometry analysis of Gr-1 and IL-4Rα in splenic CD11b-positive cells at 14 days and 28 days after cell implantation. **c** The CD8 inhibitory function assay of splenic CD11b-positive cells from 28 days after cell implantation. CFSE-labeled CD8 cells stimulated with ConA were co-cultured with splenic CD11b-positive cells obtained at 28 days after implantation for 24 h. **d** The activated CD8 T cell population changes. Splenic cells unstimulated or stimulated by CD3 and CD28 were co-cultured with intrasplenic CD11b+ cells from 14 or 28 days after cell implantation for 24 h.  − and + indicate unstimulated splenic cells and CD3/CD28 stimulated splenic cells from normal mice, respectively. *P < 0.05 and **P < 0.01 (Tukey–Kramer test). Representative data from three independent experiments that were repeated three times. One mouse per group was used 14 and 28 days after tumor implantation. One spleen of a normal mouse was used once, and the assay was repeated three times (**d**)

We further examined the morphology of intrasplenic CD11b+ cells during tumor growth. At both the early stage and late stage, intrasplenic CD11b+ cells were interspersed with neutrophils with a lobed nucleus and monocytes with an oval nucleus; it was difficult to distinguish the differences between cells at early stage and late stage by only cell morphology (Fig. 3a). Analysis of cell surface antigens revealed that intrasplenic CD11b+ cells were Gr-1^hi^, and IL-4Rα expression changed from negative to positive from the early stage to late stage (Fig. 3b). However, intrasplenic CD11b+ cells at the late stage did not show immunosuppressive activity against activated CD8 T cells (Fig. 3c, d). Based on these findings, intrasplenic CD11b+ cells were considered as MDSC-like cells (MDSC-LCs).

### TAMs exhibit unique metabolism during tumor growth

Although TAMs did not change cell surface antigens during tumor growth (Fig. 2a), TAMs showed unique immunosuppressive functions. T cell immunosuppression of TAMs has been considered to occur via Arg-1, iNOS or TNFα. In particular, the elimination of L-arginine required for T cell function by Arg-1 in both TAMs and MDSCs is one of the immunosuppressive mechanisms of these cells [36, 37]. Thus, the conversion from arginine to urea and ornithine in the urea cycle because of increased intracellular Arg-1 in both TAMs and MDSC-LCs with tumor growth may exhibit effects on various metabolic pathways. To examine intracellular metabolism changes in intratumoral TAMs and MDSC-LCs, we conducted a metabolomic analysis of these cells in the early and late stages.

**Fig. 4:**
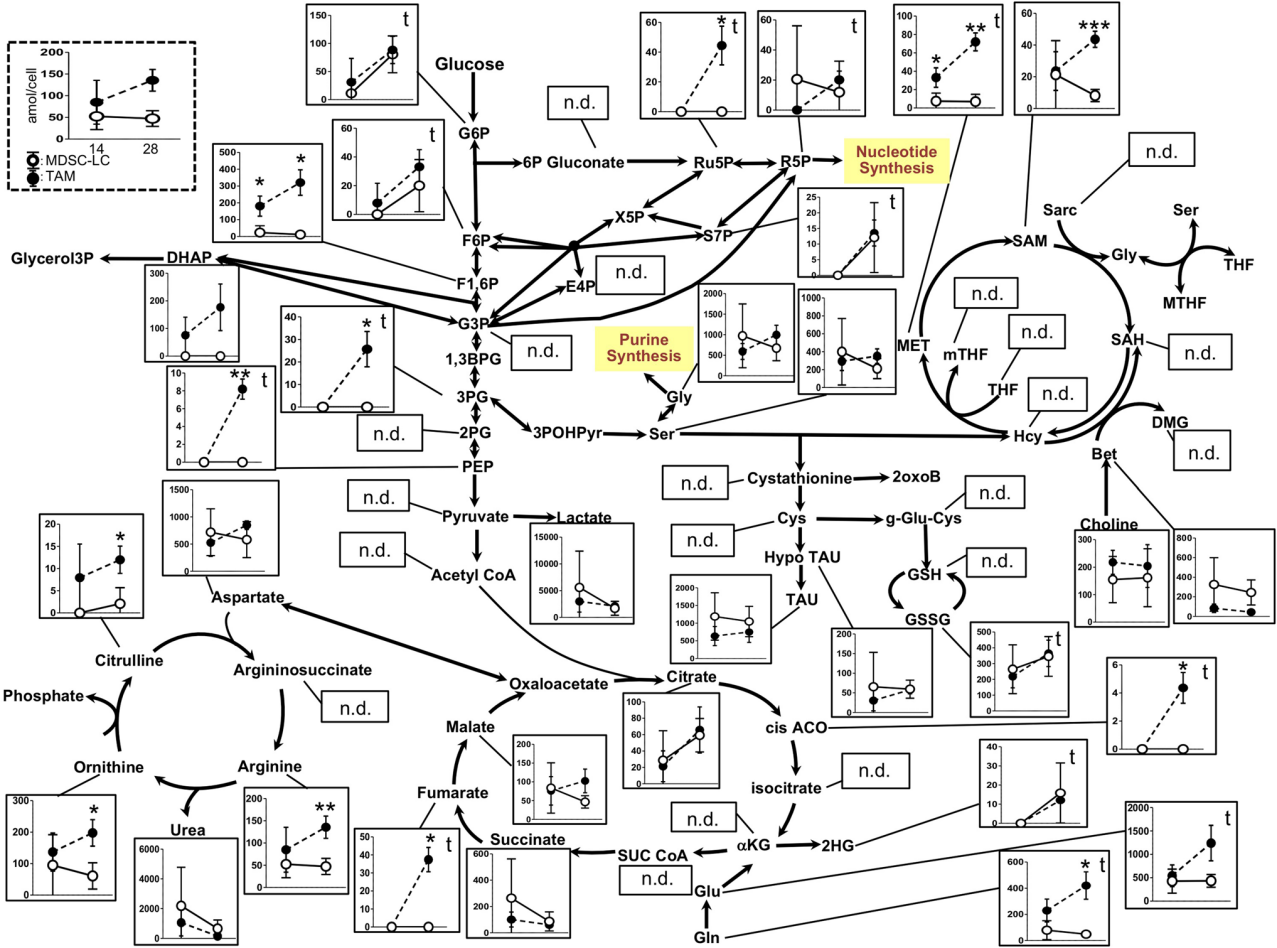
Metabolic pathway map of quantified metabolite concentrations in TAMs and MDSC-LCs during tumor growth Intracellular metabolic pathways of intratumoral CD11b+ cells classified as TAMs and splenic CD11b+ cells classified as MDSC-LCs in the early tumor stage and late tumor stage: glycolytic system (center), the tricarboxylic acid (bottom center), the methionine cycle (upper right), and the urea cycle (bottom left). The quantified concentrations are represented as mean (dot) and standard deviations (error bars). *P < 0.05 and **P < 0.01 (Student’s *t*-test between MDSC-LCs and TAMs); “t” indicates *P* < 0.05 by paired Student’s *t*-test between early and late stage of TAMs. MADS-LCs showed no significant difference between these stages. Three mice per group were used 14 and 28 days after tumor implantation

Our metabolomic analyses successfully identified and quantified various metabolites in primary pathways, such as glycolysis, TCA, PPP, urea, and one-carbon cycles (Fig. 4), and TAMs showed different metabolite concentrations compared with MDSC-LCs. For example, lactate, an end product of glycolysis, showed no differences in TAMs and MDSC-LCs. However, intermediate metabolites of glycolysis, such as glucose 6-phosphate (G6P) (*P* < 0.05), fructose 6-phosphate (F6P) (*P* < 0.05), glycerate 3-phosphate (3PG) (*P* < 0.05), and phosphoenolpyruvic acid (PEP) (*P* < 0.05), were significantly increased at the late stage compared with the early stage and only in TAMs. Interestingly, this TAM-specific trend was also observed in TCA cycle metabolites, except for succinate. Methionine (MET), *S*-adenosylmethionine (SAM), glutamine, and glutamic acid also showed a similar trend.

No metabolites in the urea cycle showed significant differences between TAMs and MDSC-LCs at the early stage, while citrulline, ornithine, and arginine showed significantly higher concentrations in TAMs compared with MDSC-LCs in late stage.

## Discussion

This study aimed to clarify how MDSCs and TAMs that work in tumor immunosuppression undergo changes in cellular metabolism during tumor growth. The morphology of tumor-infiltrating CD11b+ cells was similar to monocytes or macrophages, as the cell surface antigens were F4/80^hi^ and Gr-1^lo^. This cell population at the early stage also showed immunosuppressive function in inhibiting activated CD8 T cells. In our previous study, the intratumoral CD11b+ cells were MDSCs that exhibited M1- and M2-like macrophage properties according to the definition at the time of publication [25]. Based on the new definition of MDSCs, these cells can be defined as TAMs or tumor-infiltrating macrophages (TIMs) instead of MDSCs, because the previous study indicated that the intratumoral CD11b+ cells expressed F4/80 in 98% of the cells [38]. Therefore, the intratumoral CD11b+ cells were classified as TAMs in this manuscript. The main immune cells that secrete TNFα are macrophages. TNFα secreted from macrophages is involved in mediating various responses through TNFR1 and TNFR2 [39]. TNFα activates regulatory T cells via TNFR2 on regulatory T cells, while it induces apoptosis of CD8 T cells via TNFR2 on CD8 T cells [40]. Both direct mechanisms of activation of regulatory T cells and inhibition of CD8 T cells have been shown to impede the effect of cytotoxic T cells [40]. Our results also showed that TNFα from TAMs is one of the suppressors of tumor immunity.

We also observed holistic changes in the intracellular metabolism of TAMs with tumor growth, including (1) increased intermediate metabolites in glycolysis, (2) increased metabolites in the methionine cycle, and (3) accumulation of glutamine and glutamic acid. However, no significant changes in MDSC-LCs were observed during tumor growth. These results have helped clarify the changes in cell metabolism of TAMs during tumor growth.

In contrast to tumor-infiltrating CD11b+ cells, the intrasplenic CD11b+ cells were interspersed with neutrophils with lobulated nuclei and monocytes with oval nuclei. The cell surface antigens were Gr-1^hi^ and IL-4Rα^lo^ in the early tumor stage, and IL-4Rα expression increased from early to late tumor stage. However, neither intrasplenic CD11b+ cells at the early or late stage showed any suppressive activities on CD8 T cells. This led us to define the intrasplenic CD11b+ cells in our experiments not as MDSCs but as MDSC-LCs [38]. In the urea cycle in the intratumoral TAMs and MDSC-LCs, conversion from arginine to urea and ornithine did not change with tumor growth, respectively. Intracellular Arg-1 may increase in TAMs and MDSC-LCs, and the urea cycle may be proceeding as in normal cells. Many studies reported that Arg-1 expression is increased in TAMs and MDSCs and involved in inhibition of T cells [41, 42].

Traditionally, TAMs *in vivo* are defined as M2-like macrophages from a role in M1/M2 polarization. Several studies using intratumoral macrophages in a mouse model or histopathology in both mouse and human tumor tissue have shown that the characteristics of M1-like macrophages or characteristics of M1 and M2 overlap [43–45]. Based on these reports, when focusing on macrophages in the TME but not in the peritoneal macrophages of tumor-bearing mice, TAM function and polarity rely on the TME heterogeneity [46, 47]. TAMs also upregulate HIF1α and shift to glycolysis because of the hypoxic environment in the tumor, and HIF1α induces NO production by TAMs [46, 48]. This glycolysis shift is caused by the AKT-mTOR-HIF1α pathway [49]. Therefore, TAMs under hypoxic environments are also expected to show a shift to glycolysis. Our study also suggested that glycolysis is enhanced in intratumoral TAMs with tumor growth. Furthermore, the intermediate metabolites in the methionine cycle branching from glycolysis also increased. Although TAMs have been reported to shift to glycolysis, our study further indicates that glutamine and glutamic acid are enhanced in TAMs and flow into the TCA cycle, resulting in glutaminolysis during tumor growth. In addition, the expressions of arginase 1, arginase 2, and tryptophan-consuming enzyme indoleamine 2,3-dioxygenase 1 (IDO1) are elevated and enhanced the depletion of auxotroph (e.g., arginine, tryptophan) in the TME [50, 51]. Thus, the metabolic changes in TAMs would contribute to the escape of tumors from immunosurveillance [37, 52]. In this study, the above-mentioned intracellular metabolism changes of TAMs during tumor growth, such as these factors (e.g., Arg1 and IDO1), may occur. A previous study showed that lactic acid is accumulated in the TME, and the expression of Arg1 and VEGFRA by M2-like macrophages is increased through HIF1α activity [53, 54]. In contrast, the accumulation of lactic acid was not observed in the intracellular metabolism of TAMs during tumor growth in this study. Therefore, our data indicated that the accumulation of lactic acid in the TME may induce the immunosuppressive function of TAMs.

We initially considered intrasplenic CD11b+ cells as MDSCs in tumor-bearing mice based on cell surface antigens. However, these cells were later considered as MDSC-LCs rather than MDSCs, as the intrasplenic CD11b+ cells showed no immune suppressive function against CD8 T cells even at the late tumor stage. Because intrasplenic CD11b+ cells in this study are heterogeneous cells including monocytes and neutrophils, it will be necessary to perform finer cell sorting for distinct MDSCs, not MDSC-LCs, to determine immunosuppressive capacity and the metabolic change with tumor growth. Future studies should thus investigate changes in the immunosuppressive capacity of TAMs and MDSCs during tumor growth and clarify the association between intracellular metabolism and the immunosuppressive capacity.

In conclusion, MDSC-LCs increased with tumor growth but did not show a clear immune suppressive function like intratumoral TAMs even at the early stage and late stage of the tumor. In contrast, intratumoral TAMs distinctly showed immunosuppressive function from the early stage of the tumor via TNF-α. Regarding the intracellular metabolism of TAMs, we showed that glucose uptake increased, methionine cycle was enhanced, and glutamine and glutamic acid accumulated with tumor growth. In this study, we clarified the intracellular metabolic changes of intratumoral TAMs and MDSC-LCs associated with tumor growth. These results may lead to the development of novel immunotherapies that target intracellular metabolic changes in intratumoral TAMs.

## Data Availability

The datasets used and/or analyzed during the current study are available from the corresponding author on reasonable request.
